# General and specific components of depression and anxiety in an adolescent population

**DOI:** 10.1186/1471-244X-11-191

**Published:** 2011-12-07

**Authors:** Jeannette Brodbeck, Rosemary A Abbott, Ian M Goodyer, Tim J Croudace

**Affiliations:** 1 Developmental and Life-course Research Group, Department of Psychiatry, University of Cambridge, Douglas House, 18b Trumpington Road, Cambridge, CB2 8AH, UK

## Abstract

**Background:**

Depressive and anxiety symptoms often co-occur resulting in a debate about common and distinct features of depression and anxiety.

**Methods:**

An exploratory factor analysis (EFA) and a bifactor modelling approach were used to separate a general distress continuum from more specific sub-domains of depression and anxiety in an adolescent community sample (n = 1159, age 14). The Mood and Feelings Questionnaire and the Revised Children's Manifest Anxiety Scale were used.

**Results:**

A three-factor confirmatory factor analysis is reported which identified a) mood and social-cognitive symptoms of depression, b) worrying symptoms, and c) somatic and information-processing symptoms as distinct yet closely related constructs. Subsequent bifactor modelling supported a general distress factor which accounted for the communality of the depression and anxiety items. Specific factors for hopelessness-suicidal thoughts and restlessness-fatigue indicated distinct psychopathological constructs which account for unique information over and above the general distress factor. The general distress factor and the hopelessness-suicidal factor were more severe in females but the restlessness-fatigue factor worse in males. Measurement precision of the general distress factor was higher and spanned a wider range of the population than any of the three first-order factors.

**Conclusions:**

The general distress factor provides the most reliable target for epidemiological analysis but specific factors may help to refine valid phenotype dimensions for aetiological research and assist in prognostic modelling of future psychiatric episodes.

## Background

Depressive and anxiety symptoms often co-occur across the life-course resulting in a debate about common and distinct features of depression and anxiety emotional disorders. Both can be viewed as manifestations of a broad dimension of internalizing symptoms distinct from an externalizing dimension consisting of substance abuse, ADHD, oppositional and conduct disorders [1-5]. Various dimensional models have been proposed in order to distinguish common and distinct features of depression and anxiety and to further investigate the components of the broad internalizing factor. The well-known tripartite model [6] posits that negative affectivity is the shared component of depression and anxiety and that low positive affectivity is specific to depression and only weakly related to anxiety. Physiological hyperarousal is considered to be specific for anxiety. While there is good evidence for a general negative affectivity factor as an explanation for the overlap of depressive and anxious symptoms the role of physiological arousal is less clear and has to date been more significantly related to panic than to other anxiety disorders [7-9].

Other models have also emphasized the hierarchical structure of comorbidity between depression and anxiety [8,10]. These models acknowledge the role of an underlying general distress component which accounts for the communality of depression and anxiety symptoms as well as more specific sub-domains of depressive and anxious psychopathology which specify the unique components of both disorders over and above a general underlying distress factor. Both components are needed to fully represent the variation of depressive and anxious psychopathology.

A methodological shortcoming of previous research is that ordinal responses to questionnaires measuring common psychopathology symptoms were often treated as continuous. This can lead to attenuated estimates of correlations among indicators, particularly when there is a floor effect which is often the case in psychopathological scales in community samples. Additionally, factor analyses can yield "pseudofactors" as artefacts of item difficulty or extremeness and can generate incorrect test statistics and standard errors [11].

**Figure 1:**
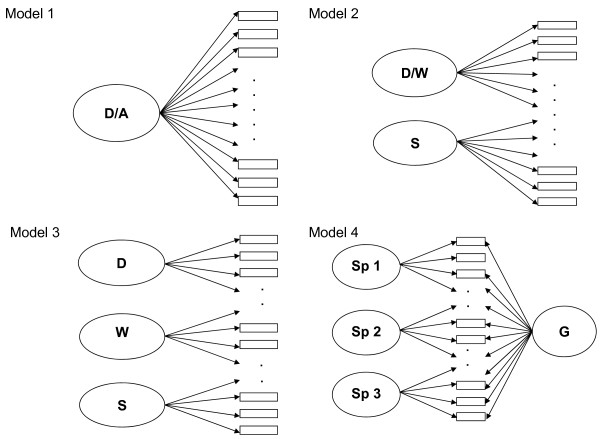
**Schematic illustration of four alternative latent variable models**. Notation: D, depression; A, anxiety; W, worrying; S, somatic symptoms; G, general distress factor; Sp1-Sp3, specific factors. Model 1, unidimensional model with one general factor; model 2, two-factor model with a depression/worrying factor and a somatic factor; model 3, three-factor model with a depression, a worrying and a somatic factor; model 4, bifactor model with a general distress factor and specific factors. Models each comprised 61 items, not all items are shown on the figures.

The purpose of the present study was to analyse common and distinct features of depression and anxiety symptoms in adolescents using self-report data from the Mood and Feelings Questionnaire (MFQ) [12], and the Revised Children's Manifest Anxiety Scale (RCMAS) [13]. Based on existing literature and exploratory factor analyses of our data, we compared a) a one factor general distress model, assuming that depression and anxiety symptoms in adolescents do not represent clearly distinguishable constructs; b) a two-factor model with one factor for cognitive and emotional symptoms of depression and anxiety, and another factor for somatic symptoms; c) a three-factor model with separate factors for depression, worrying and somatic symptoms; and d) a bifactor model, also known as a general-specific model, with a general distress factor distinguished from more specific components of depression and anxiety. These specific components account for the unique influence of the specific domains over and above the general factor and thus provide unique information completely separate from the general distress factor [14-18]. Figure 1 shows a schematic illustration of the models.

## Methods

### Participants

The sample comprised 1238 14 year-old adolescents from the ROOTs study, a British longitudinal cohort study [19,20]. Participants were recruited from Cambridgeshire schools. Twenty-seven secondary schools were approached and 18 schools agreed to take part with 3762 students invited. Response rates for individual schools ranged from 18% to 38% resulting in 33% of the adolescents taking part in the study (*n *= 1238; 46% boys and 54% girls). A total of 55% of the respondents were female and 94% were white with European origins. The socio-economic status for 14% of the sample was summarized as hard-pressed or moderate means, 24% were comfortably off, and 62% were categorised as urban prosperity or wealthy achiever. This corresponds largely to the socio-economic profile of Cambridgeshire [19]. There were no significant gender differences in ethnicity or socio-economic status.

The analysis sample included 1159 respondents (93% of the whole sample) who completed at least 85% of the MFQ and RCMAS items; 1081 had complete data on all items. The average total score was 15.33 (SD = 10.06) for the MFQ and 14.74 (SD = 10.73) for the RCMAS. Girls had higher scores on the MFQ (female mean = 17.14, SD = 10.81 vs. male mean = 13.11, SD = 8.57, t = -683, p < .000) and higher scores on the RCMAS (female mean = 17.07, SD = 11.21 vs. male mean = 11.86, SD = 9.35, t = -683, p < .000) than boys. The lifetime prevalence for an affective disorder at age 14 in the ROOTS sample was 8% and 6% for an anxiety disorder. More details about the frequency of early adversities and clinical diagnoses in the ROOTs sample can be found elsewhere [20].

The study was carried out in accordance with the Declaration of Helsinki and Good Clinical Practice guidelines. The study was approved by Cambridgeshire 2 REC, reference number 03/302. At entry into the study all participants and their parents gave written, informed consent.

### Measures

The *Mood and Feelings Questionnaire (MFQ) *is a self-report screening tool for detecting symptoms of depressive disorders in children and adolescents of 6-17 years of age [21]. MFQ items were designed to cover DSM diagnostic criteria for major depressive disorders. The scale comprised 33 items. Criterion-related validity, i.e. the ability to predict clinical diagnosis, has been established [22,23].

The *Revised Children's Manifest Anxiety Scale *(RCMAS) [13] measures general anxiety, including physiological anxiety, worry/oversensitivity, and social concerns with 28 items. An additional subscale, which was not included in this study, assessed social desirability. The assessment period for both the MFQ and the RCMAS was two weeks. The response format for both scales was modified prior to data collection to four ordered categories labelled from 0 = *never*; 1 = *sometimes*, 2 = *mostly*, to 3 = *always*. As prevalence of responses in the highest category (3 = *always*) was below 6%, the two highest categories were collapsed for further analyses (2 = *mostly and always*). Full question wording of the 61 items and response frequencies are shown in Table 1.

**Table 1:** Response frequencies of the MFQ and RCMAS items in percentages (N = 1,159)

	never	sometimes	mostly	always	missing
M_1 I felt miserable or unhappy	20.2	68.2	4.3	0.4	6.9
M_2 I didn't enjoy anything	51.9	39.5	1.6	0.2	6.9
M_3 I was less hungry than usual	45.7	34.8	10.4	2.1	7.0
M_4 I ate more than usual	41.4	42.0	7.8	1.8	7.0
M_5 I felt so tired I just sat around and did nothing	35.9	45.8	9.8	1.4	7.1
M_6 I was moving and walking more slowly than usual	65.2	23.3	3.4	1.0	7.1
M_7 I was very restless	40.2	42.0	8.7	2.1	7.1
M_8 I felt I was no good any more	69.0	20.9	2.2	1.0	6.9
M_9 I sometimes blamed myself for things that weren't my fault	60.2	27.2	4.2	1.4	7.0
M_10 It was hard for me to make up my mind	25.9	51.2	13.2	2.6	7.1
M_11 I got grumpy and cross easily	22.1	49.8	16.0	5.3	6.9
M_12 I felt like talking a lot less than usual	47.6	35.8	7.9	1.8	7.0
M_13 I was talking more slowly than usual	77.5	13.1	2.0	0.4	7.0
M_14 I cried a lot	67.8	19.6	4.3	1.3	7.0
M_15 I thought there was nothing good for me in the future	74.6	14.6	2.4	1.4	7.1
M_16 I thought that life was not worth living	79.6	10.8	1.8	0.9	6.9
M_17 I thought about dying	77.3	13.7	1.5	0.6	6.9
M_18 I thought my family would be better off without me	77.5	12.3	2.2	1.1	6.9
M_19 I thought about killing myself	84.4	7.5	0.6	0.3	7.1
M_20 I didn't want to see my friends	68.6	22.7	1.4	0.3	7.1
M_21 I found it hard to think properly or concentrate	28.0	54.8	7.9	2.2	7.1
M_22 I thought bad things would happen to me	64.2	25.6	2.2	1.0	7.1
M_23 I hated myself	71.1	17.2	3.0	1.6	7.1
M_24 I was a bad person	67.4	22.4	2.5	0.6	7.1
M_25 I thought I looked ugly	40.6	36.8	10.3	5.0	7.3
M_26 I worried about aches and pains	52.7	33.2	5.5	1.6	7.0
M_27 I felt lonely	56.8	29.6	4.8	1.8	7.1
M_28 I thought nobody really loved me	73.6	14.5	2.6	2.2	7.0
M_29 I didn't have any fun at school	50.2	34.2	5.5	2.9	7.2
M_30 I thought I could never be as good as other kids	57.3	29.2	4.4	2.0	7.1
M_31 I did everything wrong	61.1	27.6	2.8	1.4	7.1
M_32 I didn't sleep as well as usual	44.1	36.3	8.9	3.7	7.0
M_33 I slept more than usual	51.9	32.2	7.2	1.6	7.1

R_1 I had trouble making up my mind	33.1	49.0	8.2	2.2	7.5
R_2 I worried when things did not go the right way for me.	41.9	39.1	9.9	1.9	7.1
R_3 Others seemed to do things more easily than I could	33.3	45.4	11.1	3.1	7.1
R_4 Often I had trouble getting breath	74.0	16.0	2.4	0.6	7.0
R_5 I worried a lot of the time	51.4	31.2	7.5	2.8	7.1
R_6 I was afraid of a lot of things	66.9	21.3	3.5	1.1	7.2
R_7 I got angry easily	35.7	39.2	12.3	5.5	7.3
R_8 I worried about what my parents would say to me	54.3	29.7	6.7	2.2	7.1
R_9 I felt that others did not like the way I did things	47.5	37.3	6.7	1.5	7.1
R_10 It was hard for me to get to sleep at night	38.4	38.7	10.7	5.1	7.1
R_11 I worried about what other people thought about me	33.8	41.9	13.0	4.2	7.1
R_12 I felt alone even when there were people with me	66.2	21.3	3.5	1.8	7.1
R_13 Often I felt sick to my stomach	69.8	20.0	2.6	0.4	7.2
R_14 My feelings got hurt easily	50.8	31.1	7.9	3.2	7.0
R_15 My hands felt sweaty	58.5	27.1	5.1	2.2	7.2
R_16 I was tired a lot	30.2	42.6	14.5	5.8	7.0
R_17 I worried about what was going to happen	49.7	35.0	6.0	2.0	7.2
R_18 Other children were happier than me	43.1	37.9	7.7	3.9	7.4
R_19 I had bad dreams	72.1	17.4	2.6	1.0	7.0
R_20 My feelings got hurt easily when I was fussed at	64.8	20.9	4.9	1.8	7.7
R_21 I felt someone would tell me I did things the wrong way	57.6	28.5	5.1	1.4	7.5
R_22 I wake up scared some of the time	79.1	11.9	1.4	0.3	7.2
R_23 I worried when I went to bed at night	66.3	20.9	3.7	1.8	7.3
R_24 It was hard for me to keep my mind on my school work	34.2	42.7	11.2	4.7	7.2
R_25 I wiggled in my seat a lot	47.2	30.4	11.0	4.3	7.1
R_26 I worried	40.4	40.5	8.1	3.7	7.3
R_27 A lot of people were against me	68.6	19.2	3.1	1.8	7.3
R_28 I often worried about something bad happening to me	61.9	26.0	3.6	1.4	7.1

M = Mood and Feelings Questionnaire

R = Revised Children's Manifest Anxiety Scale

### Data analysis

Initial analysis of the joint item pool was conducted in stages. First, we computed exploratory factor analyses for categorical data for each scale and for pooled items under promax rotation using M*plus *[24]. A similar analysis using ULS was performed using the freeware programme FACTOR [25] which also estimates second order factor models from first-order EFA solutions, including a Schmid-Leiman decomposition of the second order factor model. Based on these results, a series of factor analyses for categorical items were specified with a single general factor and up to three specific factors (see below). To test for the generality of the models we also performed exploratory factor analyses with a random split-half sample (split1, *n *= 540). Based on these results, a series of confirmatory factor analyses on the validation sample (split2, *n *= 539). As the factor structure and the items loading on the factors were similar for the two split-half analyses and the whole sample we only report the results for the whole sample to maximize the sample size. Post-hoc modelling identified some structural refinements based on modification indices and a slightly revised model was proposed.

Thresholds and Scale Information Functions were calculated with the ordinal factor analyses procedures in M*plus*. Thresholds locate the items along the latent distress continuum according to item severity. Categorical item factor analysis in M*plus *does not report item thresholds which are directly comparable to IRT parameters. Therefore to compute the thresholds (b1 and b2) tau estimates were divided by the factor loadings [26]. The standard errors of measurement were computed from the inverse of the square root of the information function and were plotted using graphics commands. These graphs are important to provide an indication of variations in the level of estimated score precision across the measurement range and to identify the range of scale values, which are measured with highest precision.

Uniform differential item functioning (DIF) for gender was analysed in the context of a MIMIC model [11]. Uniform differential item functioning is present when items on a scale behave differently for subgroups of a population, holding the latent trait constant. This would reflect other potential influences on item responses than the underlying factor(s). As a first step, we added gender as a covariate to the models. We then fixed all the direct effects of gender on the items to zero, assuming that there is no direct effect and inspected the modification indices [11]. DIF was considered for any item with a large modification index (> .30). In a subsequent step we added a direct effect of gender on those items and inspected the change in the estimates.

Model estimation was performed using robust Weighted Least Squares (rWLS; estimator = Weighted Least Squares Mean and Variance adjusted (WLSMV)). Estimation using rWLS returns modified standard errors and a corrected chi-square test statistic of model fit. Unlike Maximum Likelihood (ML) estimation for factor analysis of continuous scores, our use of Muthén's categorical data factor analysis methodology provides asymptotically unbiased, consistent and efficient parameter estimates as well as a correct chi-square test of fit with dichotomous or ordinal observed variables. In all models individuals with partially missing item level data were included, since estimation of missing data patterns is possible under traditional ML and WLSMV.

Model fit was assessed through following different indices: the Comparative Fit Index (CFI), the Tucker Lewis Index (TLI), and the Root Mean Square Error of Approximation (RMSEA). Although no single set of threshold values for these statistics can be relied upon in isolation we favoured models that exceeded 0.95 for TLI and CFI [27-29] and models with an RMSEA approaching 0.05 [30]. To compare non-nested models, which have not a subset of the free parameters of each other and cannot be compared using χ2 difference tests, we report the sample size adjusted Bayesian Information Criteria (ssaBIC) from traditional linear factor analysis models, treating data as continuous.

Item Response Theory (IRT) informed analyses were performed to investigate the severity of symptoms by modelling how the probability of responding to an item varies as a function of the location along the underlying latent distress continuum.

## Results

### Confirmatory latent structure analysis for the first-order models

Preliminary exploratory factor analysis for ordinal data showed a reasonable model fit for a two-factor and three-factor solution. The single-factor model yielded slightly lower goodness-of-fit indices and a four-factor model resulted in factors which were difficult to interpret. In the subsequent confirmatory factor analyses for categorical data, only the three-factor model and the bifactor model fitted the data well (see Table 2). The single-factor model and the two-factor model did not achieve CFI and TLI values > 0.95.

**Table 2:** CFA-modelling results for latent structure models for MFQ and RCMAS data in adolescents aged 14

Estimator robust WLS	Chi Squ. (DF)	df	# parameters	CFI	TLI	RMSEA	WRMR	SSABIC	SSABIC +/-
1 factor model	5345.797	1764	188	0.939	0.937	0.042	1.712	104 332	0
2 factor model a^a^	4654.275	1758	194	0.951	0.948	0.038	1.565	103 636	- 696
2 factor model b^b^	5037.228	1763	189	0.944	0.942	0.040	1.647	103 839	-493
3 factor model	4083.833	1752	200	0.960	0.958	0.034	1.424	102 753	- 1579
- With gender as covariate	4248.097	1810	203	0.957	0.955	0.034	1.450	104 249	-83
- Correction for differential item functioning	4135.5731	1808	205	0.959	0.957	0.033	1.425	104 073	-259

Estimator robust WLS	Chi Squ. (DF)	df	# parameters	CFI	TLI	RMSEA	WRMR	SSABIC	SSABIC +/-

Bifactor model	3839.960	1724	228	0.964	0.962	0.033	1.350	102 077	-2255
- With gender as covariate	3951.343	1781	232	0.961	0.959	0.032	1.367	104 651^c^	+319
- Correction for differential item functioning	4083.833	1752	233	0.960	0.958	0.034	1.424		

^a^anxiety/depression and somatic factor, following EFA.

^b^two-factor model with MFQ items on one factor and RCMAS items on the other factor.

^c^computing the ssaBIC for the bifactor model with adjustments for DIF was computationally unmanageable with MLR.

SSABIC = Sample-size adjusted Bayesian information criterion.

Robust WLS is WLSMV in Mplus i.e. robust Weighted Least Squares for categorical data, mean and variance adjusted.

Robust ML is MLMV in Mplus i.e. Maximum Likelihood covariance structure analysis, mean and variance adjusted.

Model fit improved considerably when correlated errors were included for similarly worded items representing identical items/item overlap in the MFQ and the RCMAS (e.g. "*It was hard for me to make up my mind*" and "*I had trouble making up my mind" r *= .67).

The three-factor model consisted of a depressed mood factor (31 items), a worrying factor (20 items), and a somatic/information processing factor (21 items). This third factor included concentration, decision-making, irritability and somatic symptoms such as sleeping difficulties, tiredness, motor retardation and restlessness. Factor loadings of all models are presented in Table 3.

**Table 3:** Standardized loadings for the three-factor model and the bifactor model and severity parameters for the bifactor model

	three-factor model			bifactor model				Severity parameters	
**Abbreviated items**	**depression**	**worrying**	**somatic symptoms**	**general factor**	**hopelessness suicidal thoughts**	**generalized worrying**	**restlessness -fatigue**	**1. threshold**	**2. threshold**

M_1 miserable or unhappy	0.74			0.68	0.27			-1.15	2.41
M_2 not enjoy anything	0.55			0.51	0.21			0.29	4.08
M_ 3 less hungry	0.40			0.39				-0.05	2.82
M_8 no good any more	0.82			0.73	0.42			0.89	2.49
M_9 blamed myself	0.72			0.67	0.23			0.57	2.31
M_12 talking less	0.29		0.34	0.59				0.05	2.15
M_14 cried a lot	0.30	0.40		0.65	0.16			0.94	2.40
M_15 nothing good in the future	0.77			0.67	0.47			1.27	2.60
M_16 life not worth living	0.86			0.69	0.67			1.54	2.74
M_17 thought about dying	0.72			0.61	0.49			1.56	3.30
M_18 my family would be better off without me	0.77			0.65	0.52			1.48	2.77
M_19 thought about killing myself	0.80			0.66	0.56			2.03	3.50
M_20 didn't want to see friends	0.55			0.55				1.16	3.80
M_22 bad things would happen to me	0.45	0.35		0.77	0.15			0.65	2.36
M_23 hated myself	0.86			0.77	0.44			0.95	2.13
M_24 bad person	0.70			0.65	0.24			0.92	2.82
M_25 looked ugly	0.47	0.26		0.72				-0.22	1.36
M_27 felt lonely	0.78			0.72	0.30			0.39	2.04
M_28 nobody really loved me	0.82			0.72	0.44			1.13	2.25
M_29 no fun at school	0.38		0.23	0.58				0.17	2.31
M_30 never be as good as other kids	0.76			0.72	0.19			0.42	2.07
M_31 did everything wrong	0.77			0.74	0.15			0.55	2.28
R_3 others seemed to do things more easily	0.72			0.71				-0.51	1.45
R_4 trouble getting breath	0.24		0.34	0.55				1.51	3.35
R_9 others did not like the way I did things	0.74			0.73				0.04	1.85
R_12 alone even when there were people with me	0.81			0.75	0.28			0.75	2.09
R_13 sick to my stomach	0.68			0.67				1.01	2.76
R_14 got hurt easily	0.22		0.57	0.77				0.16	1.53
R_18 other children were happier	0.81			0.77	0.18			-0.12	1.49
R_27 people were against me	0.70			0.66	0.19			0.97	2.45

M_10 hard to make up mind		0.22	0.34	0.52				-1.13	1.83
M_14 cried	0.30	0.40		0.66				0.92	2.36
M_22 bad things would happen to me	0.45	0.35		0.76				0.66	2.39
M_25 looked ugly	0.47	0.26		0.72				-0.22	1.36
M_26 worried about aches and pains		0.20	0.36	0.50				0.34	2.86
R_1 trouble making up my mind		0.19	0.49	0.63				-0.59	1.92
R_2 worried when things did not go the right way		0.80		0.76		0.24		-0.16	1.50
R_5 worried a lot of the time		0.83		0.76		0.46		0.17	1.61
R_6 afraid of a lot of things		0.82		0.76		0.29		0.76	2.16
R_8 worried about what my parents would say		0.74		0.72				0.29	1.82
R_11 worried about what other people thought about me		0.75		0.73				-0.48	1.23
R_14 got hurt easily	0.22	0.57		0.77				0.16	1.53
R_17 worried about what was going to happen		0.84		0.78		0.32		0.12	1.74
R_19 bad dreams		0.56		0.55				1.38	3.24
R_20 got hurt easily when I was fussed at		0.77		0.75				0.71	1.95
R_21 someone would tell me I did things the wrong way		0.69		0.67				0.46	2.21
R_22 wake up scared		0.61		0.57		0.23		1.84	3.63
R_23 worried when I went to bed		0.70		0.63		0.43		0.90	2.48
R_26 worried		0.82		0.74		0.50		-0.22	1.54
R_28 worried about something bad happening to me		0.81		0.78		0.16		0.55	2.06

M_4 ate more			0.28	0.25				^a^	^a^
M_5 so tired I just sat around and did nothing			0.58	0.50			0.29	-0.58	2.34
M_6 moving and walking more slowly			0.55	0.47			0.33	1.13	3.55
M_7 restless			0.46	0.37			0.48	-0.46	3.24
M_10 hard to make up my mind		0.22	0.34	0.52				-1.13	1.83
M_11 grumpy and cross easily			0.70	0.62			0.18	-1.16	1.19
M_12 talking a lot less than usual	0.29		0.34	0.59				0.05	2.15
M_13 talking more slowly than usual			0.54	0.47			0.25	2.06	4.15
M_21 hard to think properly or concentrate			0.79	0.69			0.29	-0.75	1.78
M_26 worried about aches and pains		0.20	0.36	0.50			0.20	0.34	2.86
M_29 no fun at school	0.38		0.23	0.58				0.17	2.31
M_32 didn't sleep as well as usual			0.61	0.52			0.39	-0.13	2.12
M_33 slept more			0.15	0.13				^a^	^a^
R_1 trouble making up my mind		0.19	0.49	0.63				-0.59	1.92
R_4 trouble getting breath	0.24		0.34	0.55				1.51	3.35
R_7 got angry easily			0.69	0.61			0.17	-0.48	1.43
R_10 hard for me to get to sleep			0.62	0.54			0.27	-0.41	1.76
R_15 hands felt sweaty			0.48	0.42			0.20	0.79	3.38
R_16 tired a lot			0.71	0.61			0.39	-0.75	1.28
R_24 hard to keep my mind on school work			0.75	0.68				-0.50	1.40
R_ 25 wiggled in my seat			0.52	0.44			0.33	0.05	2.20

M = Mood and Feelings Questionnaire, R = Revised Children Manifest Anxiety Scale

The blanks refer to items which were not specified to load on the relevant factors in the confirmatory factor analysis.

^a ^Due to the very low factor loading < .3 on the general factor, severity parameters were not computed.

To test for a confounding effect of the different response scales (an instrument "method" effect), we included orthogonal method factors for the MFQ and the RCMAS scales. The goodness-of-fit indices and the factor structure remained similar (*χ^2 ^*= 3779.82, df = 1691, CFI = 0.96, TLI = 0.96, RMSEA = 0.03).

Inter-factor correlations were *r *= .79 for the depressed mood and worrying factor; *r *= .86 for the depressed mood and somatic/information processing factor; and *r *= .78 for the worrying and somatic/information processing factor. Some RCMAS items assessing social concerns (e.g. *"Others seemed to do things more easily than I could", "I felt that others did not like the way I did things"*) loaded substantially (> .70) on the latent depressed mood factor, but not on the worrying factor. MFQ items on the worrying factor showed only small to medium loadings (e.g. *"I thought bad things would happen to me", "I thought I looked ugly"*).

**Figure 2:**
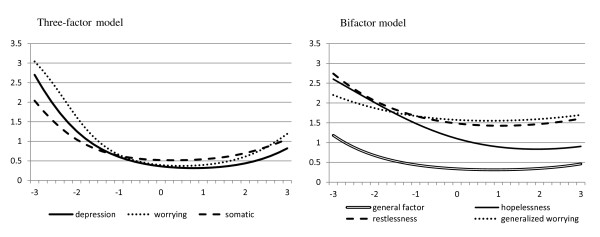
**Conditional standard error of measurement for the three-factor model and the bifactor model**. The x-axis represents the population continuum of the estimated latent trait scores in terms of a standardized normal distribution (*M *= 0, *SD *= 1).

The conditional standard errors of measurement shown in Figure 2 indicate that the measurement precision of the factors was highest around and slightly above the mean, i.e. around the population average. This declined rapidly at the lower end of the latent trait (e.g. low depression or anxiety level).

### Confirmatory latent structure analysis for the bifactor model

The bifactor model with an underlying distress factor as a general factor explained covariance among depression, anxiety and somatic symptoms [15]. The model yielded specific factors for hopelessness-suicidality, restlessness-fatigue, and generalized worrying. Although most goodness-of-fit indices suggested that the three-factor model and the bifactor model were equivalent, the sample-size adjusted BIC comparisons showed that the bifactor model (ssABIC 102,077) was favoured over the three-factor model (ssABIC 102,753, Δ -676). We caution however that these BIC values are taken from traditional linear factor models.

Table 3 presents the standardized factor loadings and IRT thresholds from the bifactor model. Almost all items had medium to large loadings on the general factor. The loadings on the specific depressed mood factor, which contained 20 items, were highest for items assessing hopelessness and suicidal thoughts (all > .49). The loadings on the specific generalized worrying factor (8 items) were highest for *"I worried"*, *"I worried a lot of the time"*, and *"I worried when I went to bed"*(loadings > .40) The specific generalized worrying factor only contained three items with factor loadings > .40, which were all similarly worded. The specific restlessness-fatigue factor had the highest loadings for restlessness (loading = .48), disturbed sleep and tiredness (both loadings = .39). The conditional standard error of measurement (see Figure 2) for the composite general distress factor increased the precision of measurement and achieved higher precision beyond the middle of the measurement scale. However the restlessness-fatigue factor and the generalized worrying factor showed a rather low precision across the whole latent trait.

### Severity of symptoms along the underlying general distress continuum

Thresholds locate the individual items along the latent distress continuum according to item severity (see Table 3). Higher threshold parameters indicate lower prevalence and higher severity on the latent distress continuum. The first threshold specifies the location on the latent distress dimension where the probability of endorsing *sometimes *becomes higher than endorsing *never*. The second threshold specifies the location on the latent distress dimension where the probability of endorsing *mostly *and *always *becomes higher than endorsing *sometimes*.

Items with higher values on the latent distress trait were related to motor retardation, suicidality, and specific night time worries. Problems with concentration and decision-making were generally located at the less severe end of the latent distress trait. A marked difference between the first ('sometimes' vs. 'never') and the second thresholds ('mostly/always' vs. 'sometimes') was found for the items *'I didn't enjoy anything*', '*I was very restless*' and '*I felt miserable or unhappy*'. Thus the 'occasional' occurrence of these symptoms was common amongst adolescents, but persistence was associated with very high severity on the underlying distress dimension.

### Gender difference and differential item functioning

The MFQ and RCMAS items did not show a gender bias for most items. Differential item functioning was found for only two items, "*I cried a lot*" and "*I thought I looked ugly*". Details are presented in Table 4.

**Table 4:** Modelling results for gender differential item functioning (male = 1, female = 2)

	Estimate	**S.E**.	**Est./S.E**.	p-value	StdY
Three-factor MIMIC model with gender as covariate					
Depression on sex	0.39	0.05	8.13	0.000	0.52
Worrying on sex	0.49	0.05	10.65	0.000	0.69
Somatic on sex	0.12	0.05	2.34	0.019	0.15

Three-factor MIMIC model with gender as covariate and direct effects for M_14 and M_25					
Depression on sex	0.36	0.05	7.58	0.000	0.49
Worrying on sex	0.46	0.05	10.08	0.000	0.65
Somatic on sex	0.12	0.05	2.39	0.017	0.15
M_14 (I cried a lot) on sex	0.91	0.09	10.18	0.000	0.91
M_25 (looking ugly) on sex	0.55	0.06	8.81	0.000	0.55

Bifactor MIMIC model with gender as covariate					
General on sex	0.32	0.04	7.39	0.000	0.47
Hopelessness-suicidal thoughts on sex	0.09	0.03	3.16	0.002	0.31
Generalized worrying on sex	0.15	0.03	5.03	0.000	0.59
Restlessness-fatigue on sex	-0.12	0.03	-4.07	0.000	-0.42

Bifactor MIMIC model with gender as covariate and direct effects for M_14 and M_25					
General on sex	0.28	0.04	6.42	0.000	0.41
Hopelessness-suicidal thoughts on sex	0.11	0.03	3.62	0.000	0.37
Generalized worrying on sex	0.18	0.03	5.62	0.000	0.70
Restlessness-fatigue on sex	-0.09	0.03	3.62	0.000	-0.30
M_14 (I cried a lot) on sex	0.97	0.09	10.83	0.000	0.97
M_25 (looking ugly) on sex	0.65	0.06	10.47	0.000	0.65

Thus, the underlying structure of these factors was similar in boys and girls and the differences in overall symptom level between males and females were not affected by DIF. Therefore in the three-factor model, the considerably higher means on the depressed mood and the worrying factor and the slightly higher score on the somatic/information processing factor among girls can be attributed to real differences in these factors and not to gender bias. Similarly, DIF did not account for the gender differences in the bifactor model where girls had higher scores on the general distress factor, the hopelessness-suicidal thoughts and the generalized worrying factor, but lower scores in the restlessness-fatigue factor.

## Discussion

This study investigates general and specific features of self-reported depression and anxiety in adolescents. Alternative factor models to characterise the latent structure of depression and anxiety symptoms as IRT-informed dimensional phenotypes using latent trait modelling principles and methods were compared. In our large sample of British 14-year-old adolescents a three-factor model was preferred over one or two factor solutions in initial EFA. The three-factor (first-order) model contained a depressed mood factor, consisting of affective and social-cognitive symptoms of depression, a worrying factor, as well as a somatic/information processing factor including psychomotor disturbance, irritability, and thinking/decision-making difficulties. Under this model these factors can be viewed as distinct yet closely related constructs. Alternatively, a bifactor model representation also fitted the data well. This representation is in line with recent theoretical developments and offers improved insights into specific factors.

The *three-factor model *reflects the view that depression and anxiety show a clearly distinguishable symptomatology. The distinct somatic/information processing factor implies that symptoms including concentration, irritability, sleeping difficulties, tiredness, and motor disturbances to be at the same hierarchical level with the depressed mood and the worrying factor, rather than being a subordinate construct. This is in line with structural studies of adult self-report depression scales which yield cognitive and somatic factors [31]. In contrast to the tripartite model, the somatic/information processing factor in the three-factor solution in this study of adolescents contains not only arousal symptoms, but also psychomotor retardation, decision making and concentration difficulties.

Although the fit indices of the three-factor model were good, the substantial correlations of the factors suggest an alternative interpretation in terms of a common dimension for depressive, anxious, and somatic symptoms-a general factor influencing all items. Our *bifactor model *formulation, which is based on the initial Mplus and FACTOR results, supports the hypothesis of a *general distress factor *for depression and anxiety which accounts for a large proportion of the communality of depression and anxiety items and is consistent with an internalizing factor with depression, generalized anxiety disorder, and social anxiety [32,3-5]. The bifactor model confirmed reliable variance for two domain specific factors for hopelessness-suicidality and restlessness-fatigue respectively. As expected, given the number and magnitude of item loadings, the general distress factor shows higher measurement precision and allows more precise measurement across a broader range of the population continuum than the specific factors and the three-factor (first order) model.

For these reasons, the bifactor representation proved to be more useful as a model for the structure of depression and anxiety symptoms in adolescents than the three factor model.

Our findings highlight the importance of domain specific factors which provide unique information over and above the general distress factor and reflect the distinctiveness of certain symptomatology and illness signs within depression and anxiety. The most salient features of psychopathology in the domain specific factor are hopelessness and suicidal thoughts, contrary to low positive affect or anhedonia as described by the tripartite model. Importantly, this *hopelessness-suicidality factor *capturing a distinct feature of depression is associated with a higher severity on the latent distress continuum. In a similar framework applied to adult data, Simms et al. [10] found that suicidality, panic, appetite loss, and ill temper were associated with higher levels on the underlying distress dimension. Low well-being, generalized anxiety, lassitude, and dysphoria were associated with lower levels of distress. Few studies have attempted general-specific factor separation in adolescents.

The specific *restlessness-fatigue factor *is analogous to somatic-endogenous constructs used clinically. It does not include items assessing other physiological symptoms such as shortness of breath or sweaty hands and is therefore distinct from the hyperarousal factor of the tripartite model.

The specific factor for *generalized worrying *contained only three items with factor loadings > .4, which were all similarly worded. Therefore, the relationship among these items could potentially represent a methodological artefact, able to be modelled using correlated errors rather than a specific psychopathological worrying factor. Thus, in a school-based community sample of adolescents, anxious symptoms seem more to be associated with general distress than reflecting a specific psychopathological construct. This view makes the bifactor representation more parsimonious, since it suggests only two specific factors.

A *limitation *of these results is that only self-report data were included in our cross-sectional analysis of the baseline phase of an ongoing longitudinal study. Longitudinal data are essential to further examine stability in the general and the specific factors over time. External correlates may help to elucidate potential aetiological factors. In addition, the anxiety self-report measure used is relatively weak on ascertaining fear based items and contains relatively few items specific for obsessional and compulsive acts that can be correlated with anxiety. This may account for the lack of validity in the specific worry factor. A further limitation is the relatively low response rate to initial recruitment within schools. This could be due to the ethically approved recruitment strategy which required participants to actively "opt in" rather than "opt out". We were aware that highly dysfunctional families could form a higher proportion of families that did not actively opt in to the study. Finally, factor structures and gender effects might differ according to the degree of psychopathology. This possibility needs to be explored in suitably large clinical samples.

## Conclusions

The general distress factor, underlying depression and anxiety items, provides a reliable target for epidemiological analysis. The specific factors for hopelessness-suicidal thoughts and restlessness-fatigue may help to refine valid phenotype dimensions, and assist in prognostic modelling of future psychiatric episodes. Furthermore, the role of aetiological factors such as genotype, early adversities, or intermediate psychoendocrine phenotypes can be investigated independently for the general and specific factors, which may improve our understanding of putative subtypes within common emotional mental illnesses. Implications for future research are to promote building groups with general or specific factors for different domains which may lead to more accurate results than merely distinguishing groups by heterogeneous diagnoses.

Our results support the view that depression and anxiety disorders could be linked together in the DSM-V and ICD-11 in a more general category of emotion disorders [33]. They also support the development of intervention models which target shared aspects of depressive and anxiety disorders but also tailor treatments to address disorder specific features, revealed here by the bifactor model.

## Competing interests

The authors declare that they have no competing interests.

## Authors' contributions

JB performed the statistical analysis and drafted the manuscript. RAA contributed to the statistical analysis and the manuscript. IMG conceived and designed the study and contributed at all stages of both the study and manuscript. TJC participated in the design of the study, oversaw the analytical strategy, and contributed to the manuscript. All authors read and approved the final manuscript.

## Pre-publication history

The pre-publication history for this paper can be accessed here:

http://www.biomedcentral.com/1471-244X/11/191/prepub
